# Comparative cytogenetics in the genus *Hoplias* (Characiformes, Erythrinidae) highlights contrasting karyotype evolution among congeneric species

**DOI:** 10.1186/s13039-015-0161-4

**Published:** 2015-07-30

**Authors:** Ezequiel Aguiar de Oliveira, Luiz Antônio Carlos Bertollo, Cassia Fernanda Yano, Thomas Liehr, Marcelo de Bello Cioffi

**Affiliations:** Universidade Federal de São Carlos, Departamento de Genética e Evolução, São Carlos, SP Brazil; SEDUC-MT, Cuiabá, MT Brazil; Jena University Hospital, Friedrich Schiller University, Institute of Human Genetics, Kollegiengasse 10, D-07743 Jena, Germany

**Keywords:** Trahiras, Fish cytogenetics, FISH, Repetitive DNA, Chromosome change and speciation

## Abstract

**Background:**

The Erythrinidae fish family contains three genera, *Hoplias, Erythrinus* and *Hoplerythrinus* widely distributed in Neotropical region. Remarkably, species from this family are characterized by an extensive karyotype diversity, with 2n ranging from 39 to 54 chromosomes and the occurrence of single and/or multiple sex chromosome systems in some species. However, inside the *Hoplias* genus, while *H. malabaricus* was subject of many studies, the cytogenetics of other congeneric species remains poorly explored. In this study, we have investigated chromosomal characteristics of four *Hoplias* species, namely *H. lacerdae, H. brasiliensis, H. intermedius* and *H. aimara.* We used conventional staining techniques (C-banding, Ag-impregnation and CMA_3_ -fluorescence) as well as fluorescence *in situ* hybridization (FISH) with minor and major rDNA and microsatellite DNAs as probes in order to analyze the karyotype evolution within the genus.

**Results:**

All species showed invariably 2n = 50 chromosomes and practically identical karyotypes dominated only by meta- and submetacentric chromosomes, the absence of heteromorphic sex chromosomes, similar pattern of C-positive heterochromatin blocks and homologous Ag-NOR-bearing pairs. The cytogenetic mapping of five repetitive DNA sequences revealed some particular interspecific differences between them. However, the examined chromosomal characteristics indicate that their speciation was not associated with major changes in their karyotypes.

**Conclusion:**

Such conserved karyotypes contrasts with the extensive karyotype diversity that has been observed in other Erythrinidae species, particularly in the congeneric species *H. malabaricus.* Nevertheless, what forces drive such particularly different modes of karyotype evolution among closely related species? Different life styles, population structure and inner chromosomal characteristics related to similar cases in other vertebrate groups can also account for the contrasting modes of karyotype evolution in *Hoplias* genus.

## Background

Erythrinidae is a small family of freshwater fishes composed by three genera, *Hoplias* Gill 1903, *Erythrinus* Scopoli 1777 and *Hoplerythrinus* Gill 1895 [1]. Its species are characterized by a remarkable karyotype diversity with 2n ranging from 39 to 54 chromosomes and the occurrence of single and multiple sex chromosome systems in some species. Thus, they represent an interesting and suitable model to investigate the process of chromosomal evolution among fishes [2–5].

Although small, the actual diversity, systematics and corresponding taxonomic construction of Erythrinidae is still not well resolved. Especially in the *Hoplias* genus, three major groups of species were identified based on their morphological characters: *H. lacerdae, H. malabaricus* and *H. macrophtalmus* [6]. The *lacerdae* group was recently revised and 5 valid species are now recognized namely *H. lacerdae, H. intermedius, H. brasiliensis, H. curupira* and *H. australis,* the last two being newly described ones [6]. Species of *macrophthalmus* group have also been revised and only *H. aimara* is now recognized [7]. In turn, the *malabaricus* group still requires a taxonomic revision [3].

In fact, the overall cytogenetic data suggest that *H. malabaricus* presents an extensive karyotype variation characterized by 07 major karyomorphs easily distinguishable from each other [2, 3]. In addition, different classes of repetitive DNAs also provided relevant data about population diversification, demonstrating that they are good chromosomal markers to detect recent evolutionary events (reviewed in [4]).

Excluding *H. malabaricus*, little cytogenetic information is available for other *Hoplias* species. Some previous data points towards one similar karyotype, with an invariable 2n = 50 and the absence of differentiated sex chromosomes [8–10]. Thus, such conserved karyotypes contrast with the extensive chromosome diversity that has been observed in other Erythrinidae species, and particularly among the representatives of the *H. malabaricus* group*.*

Therefore, this study aimed to complete cytogenetic data for another rather neglected *Hoplias* species using conventional and molecular cytogenetic methods. It was aimed to (1) enhance the knowledge of the karyotype structure of these species (2) investigate the chromosomal relationships among them and (3) highlight the contrasting evolutionary pathways inside *Hoplias* genus.

## Results

**Fig. 1:**
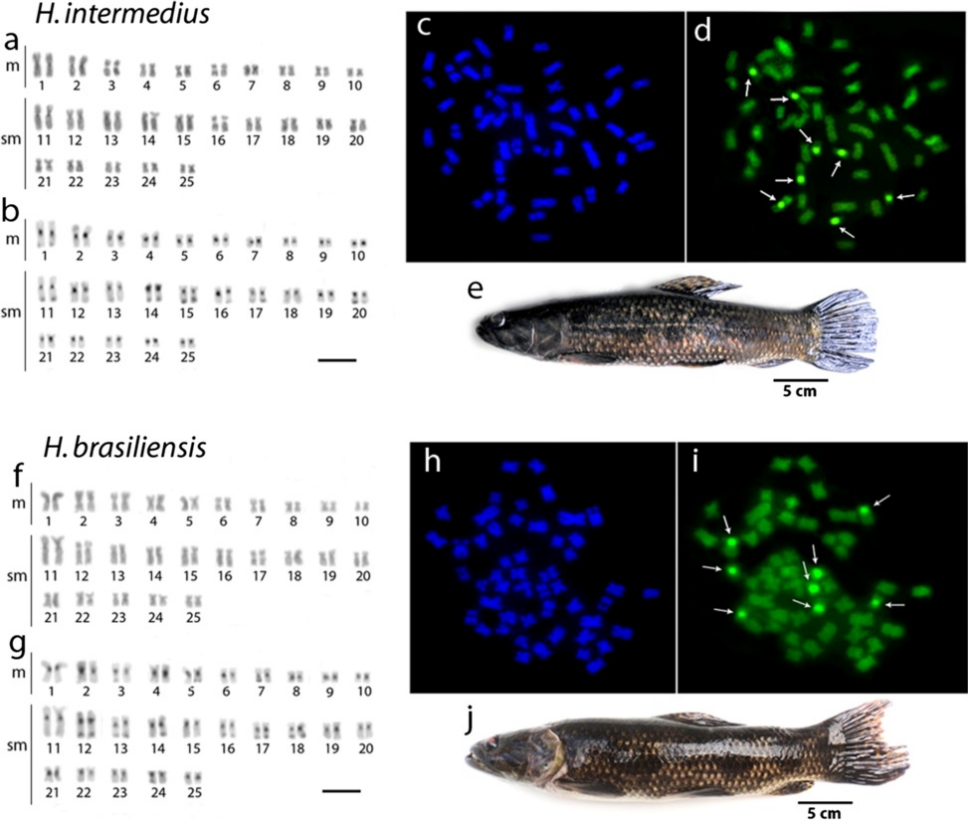
Karyotypes of *Hoplias intermedius* and *Hoplias brasiliensis* arranged from Giemsa-stained (**a**, **f**) and C-banded (**b**, **g**) chromosomes. Sequentially DAPI- (**c**, **h**) and CMA3-(**d**, **i**) stained metaphase chromosomes of both species documenting the GC-rich positive heterochromatic blocks (*arrowed*). Images from *H. intermedius* and *H. brasiliensis* are represented in (**e**) and (**j**), respectively. Bar = 5 μm

**Fig. 2:**
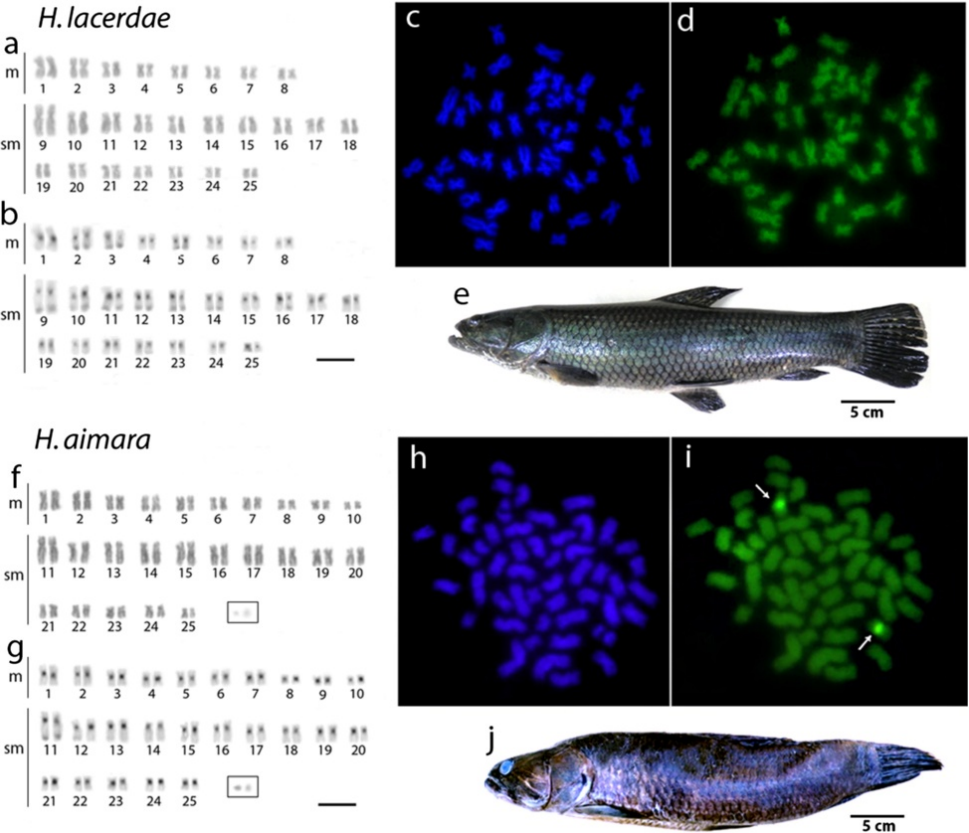
Karyotypes of *Hoplias lacerdae* and *Hoplias aimara* arranged from Giemsa-stained (**a**, **f**) and C-banded (**b**, **g**) chromosomes. Sequentially DAPI- (**c**, **h**) and CMA3-(**d**, **i**) stained metaphase chromosomes of both species documenting the GC-rich positive heterochromatic blocks (*arrowed*). Images from *H. lacerdae* and *H. aimara* are represented in (**e**) and (**j**), respectively. Bar = 5 μm Bar = 5 μm

All species under study had invariably 2n = 50 chromosomes in both sexes, showing only minor variations in their karyotypes. They possessed 20 m and 30 sm chromosomes in *H. intermedius, H. brasiliensis* and *H. aimara,* while 16 m and 34 sm chromosomes were found in *H. lacerdae*. Additionally, 1–2 B-chromosomes were presented in some *H. aimara* individuals (Figs. 1 and 2). Blocks of C-positive heterochromatin were observed in the centromeric region of all chromosomes and in the terminal region of some chromosome pairs (Figs. 1 and 2).

However, CDD (CMA_3_/DAPI) staining revealed significant variation of the fluorescent chromosomal pattern among species. While *H. intermedius* and *H. brasiliensis* showed eight CMA_3_^+^ sites at the centromeric regions of four chromosome pairs, *H. aimara* showed only 2 sites in one sm chromosome pair, also present in the two former species (Figs. 1 and 2). Contrary, *H. lacerdae*, did not present any CMA_3_^+^ sites on their chromosomes (Fig. 2).

**Fig. 3:**
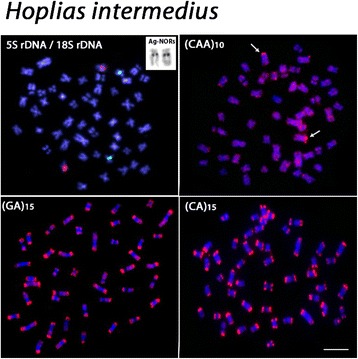
*Hoplias intermedius* chromosomes showing the 18S rDNA (red) and 5S rDNA (green) sites, the Ag-NOR bearing chromosome pair, and the distribution of (CAA)_10_, (GA)_15_ and (CA)_15_ microsatellites. Note the general distribution pattern of microsatellites and a more conspicuous (CAA)_10_ site in the short arms of a submetacentric chromosome (*arrows*). Bar = 5 μm

**Fig. 4:**
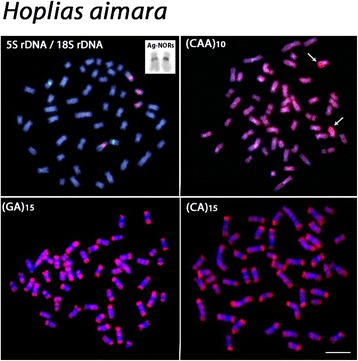
*Hoplias aimara* chromosomes showing the 18S rDNA (red) and 5S rDNA (green) sites, the Ag-NOR bearing chromosome pair, and the distribution of (CAA)_10_, (GA)_15_ and (CA)_15_ microsatellites. Most conspicuous (CAA)_10_ sites in the long arms of a submetacentric chromosome pair are indicated by arrows. Bar = 5 μm

**Fig. 5:**
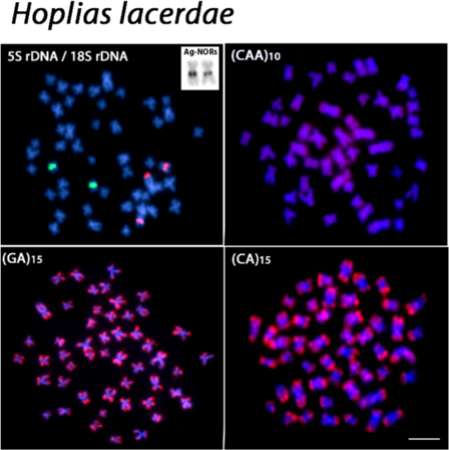
*Hoplias lacerdae* chromosomes showing the 18S rDNA (red) and 5S rDNA (green) sites, the Ag-NOR bearing chromosome pair and the distribution of (CAA)_10_, (GA)_15_ and (CA)_15_ microsatellites. Bar = 5 μm

**Fig. 6:**
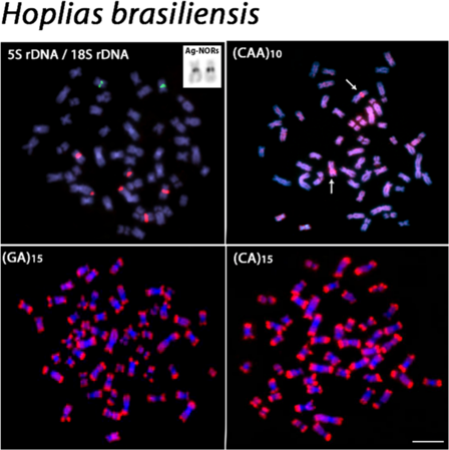
*Hoplias brasiliensis* chromosomes showing the 18S rDNA (red) and 5S rDNA (green) sites, the Ag-NOR bearing chromosome pair, and the distribution of (CAA)_10_, (GA)_15_ and (CA)_15_ microsatellites. Most conspicuous (CAA)_10_ sites in the proximal region of a metacentric chromosome pair are indicated by arrows. Bar = 5 μm

The chromosomal mapping using microsatellite DNAs (GA)_15_ and (CA)_15_ showed similar patterns in the examined species, with scattered signals and a remarkable accumulation in the subtelomeric regions of all chromosomes. However, different patterns were observed among species after FISH with the microsatellite (CAA)_10_. Besides the presence of scattered signals, a considerable accumulation of this sequence was detected in a non-homologous chromosome pair among species; with the exception of *H. lacerdae* where such accumulation was not verified (Figs. 3, 4, 5 and 6).

All species possessed only one, likely interspecifically homologous chromosome pair bearing 5S rDNA sites. Differently, the 18S rDNA sites showed species-specific pattern, representing a good cytotaxonomic marker. All species shared one homologous sm chromosome pair bearing 18S rDNA sites, corresponding also to silver-positive, active Ag-NORs. In addition, *H. brasiliensis, H. aimara* and *H. lacerdae* had four, two and one additional 18S rDNA sites, respectively, some of them homologous among these species (Figs. 3, 4, 5 and 6).

**Fig. 7:**
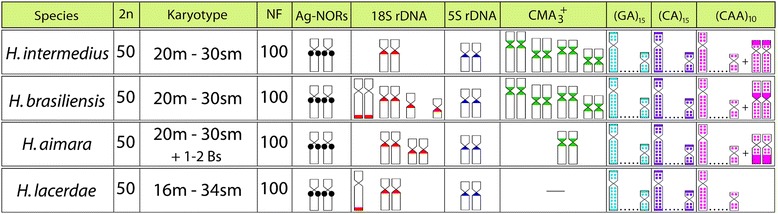
Summary of the main chromosomal characteristics of the *Hoplias* species under study

Figure 7 graphically depict the results of conventional and molecular cytogenetic analyses.

## Conclusions

### Insights into the karyotype evolution in *Hoplias*

Apart from some minor characteristics, such as a slightly different number of m and sm chromosomes in *H. lacerdae* and the presence of some B-chromosomes in *H. aimara*, the four species showed a high similarity in their karyotypes. In addition, no heteromorphic sex chromosomes were detected and a similar pattern of C-positive heterochromatin was also observed. Therefore, the speciation process in *H. lacerdae, H. brasiliensis, H. intermedius* and *H. aimara* was not accompanied by significant changes at the chromosomal level. Such conservative pattern is also supported by some previous results, where different populations of *H. aimara* and *H. intermedius* [10], and undetermined individuals of the so called “*lacerdae* group” [9], shared the same karyotypes.

However, even though obvious major changes in the karyotypes of these *Hoplias* species were not observed, the cytogenetic mapping of different repetitive DNA sequences provided reliable chromosomal markers, revealing some species-specific differences, as also reported for other different populations of *H. aimara* and *H. intermedius* [10]. In fact, repetitive DNAs are highly dynamic throughout evolution and, therefore, their application in evolutionary studies provides significant contributions [11, 12]. Although the 5S rDNA sites showed an identical position in the chromosomes of the four species, the distribution of the 18S rDNA and corresponding CMA_3_^+^ sites had different patterns. With the exception of *H. lacerdae,* all other species shared a likely identical sm chromosome pair bearing 18S rDNA/CMA_3_^+^ sites, which were also the only active Ag-NOR sites (Figs. 1, 2, 3,4, 5, 6 and 7). In fact, the cytologically detectable correspondence of GC-rich DNA with major rDNA sites is evolutionary conserved for all Actinopterygii, except Acipenseriformes [13–17]. However, not all rDNA sites were necessarily GC-rich, a condition that has also been found in other fish species [18, 19].

Additional 18S rDNA sites were found in the genome of *H. brasiliensis, H. aimara* and *H. lacerdae,* each species presenting a particular pattern of six, four and three sites, respectively. Notably, just one chromosome of the homologous pair displaying an rDNA site was observed in *H. brasiliensis* and *H. lacerdae* (Fig. 7). This pattern may be due to i) a limitation of the FISH technique in detecting the rDNA sequences in both chromosomes due to their reduced copy number; ii) the occurrence of unequal crossing overs changing the rDNA amount between homologues, iii) the simple deletion of this segment; iv) a polymorphic condition or v) the mobility of rDNA sequences by the activity of Transposable Elements (TEs). In fact, fish genomes contain many types of TEs and a number of studies have recently evidenced their potential to cause rDNA mobility [12, 20, 21]. Particularly, in another Erythrinidae species, *E. erythrinus*, the insertion of the retrotransposable element *Rex3* into rDNA sequences is thought to be the main source of the rDNA spreading in the genome [22, 23].

Microsatellites are abundant repeated sequences present in all eukaryotes studied thus far and they are found either between the coding regions of structural genes or between other repetitive sequences [24]. In fish genomes, microsatellites are usually localized in the telomeres and centromeres, where a significant fraction of repetitive DNA is also present [25]. Microsatellites (CA)_15_ and (GA)_15_ showed a general similar distribution in the four species analyzed, being abundantly located in the subtelomeric regions of all chromosomes, as also observed in some other fish species such as *Triportheus trifurcatus*, *Imparfinis schubarti, Danio rerio* and in *H. malabaricus* [26–28]. In contrast, the microsatellite (CAA)_10_ showed a specific pattern for *H. intermedius, H. brasiliensis* and *H. aimara*. Indeed, besides its wide scattered distribution along chromosomes, a strong accumulation was found in a particular chromosome pair for each species, with exception for *H. lacerdae*, therefore displaying specific zones of accumulation and pointing out distinct evolutionary pathways concerning the genome organization among *Hoplias* species.

Indeed, repetitive DNAs are characterized by a dynamic evolutionary process [29, 30] and one of the main properties of microsatellite sequences is their capacity to originate variations with different numbers of repeats [31]. In this way, the repetitive fraction of the genome (as here exemplified by the rDNAs and microsatellites) seems to escape the selective pressure that acts in the non-repetitive segments, thus being able to show recent evolutionary events [32]. Therefore, although preserving a similar karyotype, some inner chromosomal differentiations can be found among species, probably due to a restricted gene flow, thus corroborating their recent taxonomic recognition.

**Fig. 8:**
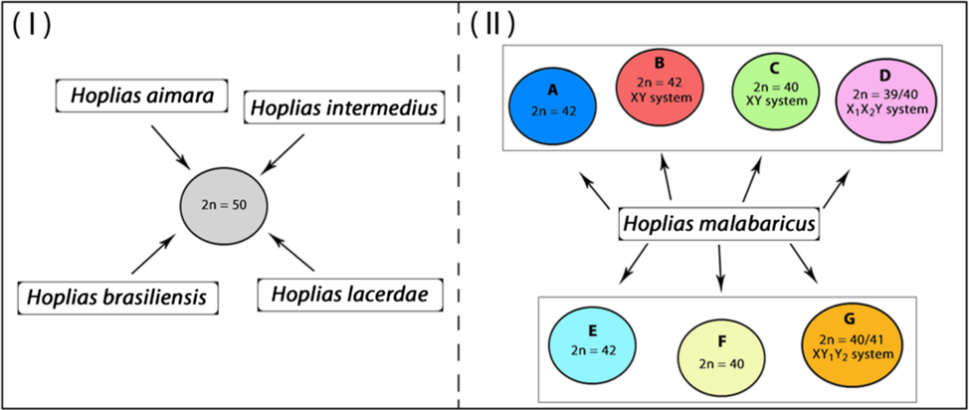
Conservative karyotype characteristics (I) shared by different *Hoplias* species contrasted with highly divergent karyotypes (II) displayed by representatives of *H. malabaricus* group, with seven major karyomorphs (A-G). In (II), boxed karyomorphs share morphological chromosomal characteristics which differ between the two boxed sets

However, the most intriguing feature emerges when we analyze the existing pattern in *H. malabaricus* (Fig. 8)*.* Differently from the karyotypes composed by 2n = 50 m and sm chromosomes conserved between sexes and species analyzed in the present study, *H. malabaricus* presents a remarkable differentiation between populations distributed throughout the Neotropics. For these populations, several karyomorphs are characterized, diverging in their 2n, karyotypes, distribution of repetitive DNA sequences and presence of simple or multiple sex chromosome systems [2–4, 33]. While some of these karyomorphs are endemic to a certain watersheds, other ones have a wide geographical distribution, being also found in sympatry without detection of hybrids [2]. In addition, molecular phylogenetic analyzes have also highlighted the evolutionary divergence within a same karyomorph, thus supporting the hypothesis that it may contain more than one species [34]. Accordingly, differentiation between populations of a single major karyomorph has also been evidenced by the distribution of repetitive DNAs on chromosomes, revealing the actual systematic diversity present in this group [35].

Therefore, a general chromosomal conservatism found in the four *Hoplias* species analyzed contrasts with the extensive karyotype diversity that has been observed in other Erythrinidae species, notably in the congeneric species *H. malabaricus.*

### What drives distinct modes of karyotype evolution among closed related species?

Karyotype diversification processes and morphological patterns are often indicators of the lifestyle of a species [36], and several vertebrates provide an opportunity to search for such correlations once some lineages have experienced faster rates of evolutionary changes in anatomy and in their way of life than others have [37].

In mammals, for example, some related taxa present distinct rates of chromosomal evolution and this might be explained by the way the species are socially organized [38]. It has been suggested that the social systems evolved by some mammalian groups produce population structures that enhance inbreeding and genetic drift, thereby facilitating the fixation of chromosomal rearrangements [39]. The key factor involved seems to be the type of social behavior, which produces small effective population sizes and inbreeding [40–43]. Interestingly, two modes of chromosomal evolution are present among salmonid fishes, which are either anadromous or found in specialized lacustrine environments. It was proposed that selection for increases or decreases in genetic recombination could have been involved in the evolution of chromosome number in these fishes, and if the rearrangements occur without a selective advantage, extensive changes would be associated with small effective population sizes [44]. Similarly, in *Hoplias* species two main situations can be also occur concerning their ecological habits. While *H. malabaricus* is characterized by having a more sedentary habitat, inhabiting mainly marginal lakes; *H. lacerdae*, *H. aimara*, *H. brasiliensis* and *H. intermedius* occupy preferably the main channel of the rivers [10]. Therefore, smaller and more isolated populations can be commonly found in *H. malabaricus*, increasing the probability of fixation of chromosomal rearrangements and thus generating intra- and inter karyotype diversity, unlike other *Hoplias* species. The different lifestyle models and population structure found within genus *Hoplias* can, therefore, be correlated with the highly differentiated chromosomal diversity among its congeneric species.

In fact, chromosomal rearrangements may spread to fixation in small populations where there is a higher probability in generating homozygous rearranged forms that are free of meiotic segregation problems [45]. The fact that many species differ by fixed and specific chromosome rearrangements, suggests that those ones that contribute to speciation are most likely to accumulate in allopatry or under restricted gene flow [46]. Indeed, fish species characterized by higher mobility and population density usually present few chromosome rearrangements, as exemplified by some migratory Neotropical fishes, such as Anostomidae, Prochilodontidae, Curimatidae, in which none or little changes are found in their karyotypes [47, 48]. On the contrary, high karyotype variability is usually present in fish groups with low mobility and exhibiting small isolated populations. Such behaviors contributed, for example, to the large chromosome variation found among fishes of the genus *Channa*, in which the diploid number varies from 2n = 32 in *C. punctata* to 2n = 112 in *C. gachua*. In this case, Robertsonian rearrangements, pericentric inversions and polyploidy characterize different species/populations, appearing as the main sources of such chromosomal diversity [49–51].

Additionally, other features related to the own genomic organization may be also taken into account in generating chromosome variability, as well exemplified in Cricetidae mammals. In this family, two lemmings’ genera, *Lemmus* and *Dicrostonyx,* have similar population structures, but while little chromosome variability is present in the former one, a higher rate is found in the later [52]. In fact, chromosome breakage in evolution is a nonrandom process, resulting in segments that are conserved over millions of years in contrast to other unstable regions that are more likely to be involved in rearrangements, because of their underlying sequence features [53]. Chromosome fragility has been also linked with the karyotype evolution of some other mammalian species, such as the rock wallabies of the *Petrogale* genus [54], primates of the *Cebidae* family [55] and deer species of *Mazama* genus [56]. Therefore, besides the different life styles, inner chromosomal characteristics may also account for the contrasting evolutionary models that occur in the *Hoplias* genus. However, some other approaches such as intrinsic molecular and meiotic features, and external issues like the effective population size, gene flow and population dynamics, will be useful tools for further clarifying this peculiar scenario.

## Methods

### Material

Chromosome preparations were obtained from four *Hoplias* species as specified in Table 1. The samples were collected with the authorization of the Brazilian environmental agency ICMBIO/SISBIO (License number 48628–2). All species were properly identified by Prof. Dr. Oswaldo T. Oyakawa, being deposited in the Museum of Zoology of the University of São Paulo (MZUSP) (Table 1).

**Table 1:** Collection sites of *Hoplias* species, with the respective sample sizes and museum codes identification

Species	Locality	Number	Museum deposit
*Hoplias brasiliensis*	Grão Mogol (MG) – Itacambiruçú River (Jequitinhonha River Basin)	08♂ 02♀	EAO2014103101
*Hoplias aimara*	Querência (MT) – Xingu River (Amazon River Basin)	03♂ 01♀	EAO2014080302
*Hoplias intermedius*	Fish culture facility (Poço Fundo – MG)	04♂ 04♀	EAO2014082801
*Hoplias lacerdae*	Fish culture facility (Poço Fundo – MG)	02♂ 06♀	EAO2014082802

*MT* Mato Grosso, *MG* Minas Gerais States

### Mitotic chromosome preparations

The animals were first injected in the abdominal region with a 0.025 % aqueous solution of colchicine at a dose of 1 ml/100 g of weight. After 50–60 min, the specimens were anesthetized and sacrificed, and the chromosomal preparations were made from cells of the anterior kidney [8]. The procedures were performed in accordance with the Ethics Committee on Animal Experimentation of the Universidade Federal de São Carlos (Process number CEUA1853260315).

### Chromosome staining

In addition to the conventional Giemsa method, chromosomes were analyzed after silver nitrate staining [57] in order to visualize the nucleolar organizing regions (Ag-NORs). C-banding was also employed to detect the C-positive heterochromatin [58] and Chromomycin A3 (CMA_3_) staining to identify the GC-rich regions on the chromosomes [15].

### Fluorescence *in situ* hybridization (FISH)

Two tandemly arrayed rDNA sequences isolated from the genome of *H. malabaricus* were used. The first probe contained a 5S rDNA repeat copy and included 120 bp of the 5S rRNA transcribing gene and 200 bp of the non transcribed spacer (NTS) [59]. The second probe corresponded to a 1400-bp segment of the 18S rRNA gene obtained *via* PCR from nuclear DNA [35]. The 18S rDNA probe was labeled with biotin-14-dATP using the kit Biotin-Nick Translation Mix (Roche), while the 5S rDNA probe was labeled with digoxigenin-11-dUTP, using the kit DIG-Nick Translation Mix (Roche), according to manufacturer’s instructions. Additionally, oligonucleotide probes containing microsatellite sequences (CA)_15_, (GA)_15_ and (CAA)_10_, directly labeled with Cy3 during synthesis by Sigma (St. Louis, MO, USA) [60], were also applied.

### Slides preparation, hybridization and signal detection

The FISH method was conducted as follows: slides with fixed chromosomes were maintained at 37 °C for 1 h. Subsequently, they were incubated with RNAse (10 mg/ml) for 1 h at 37 °C in a moist chamber. Next, it was performed a 5-min wash with 1xPBS and 0.005 % pepsin was applied to the slides (10 min at room temperature). The slides were then washed again with 1xPBS. The material was fixed with 1 % formaldehyde at room temperature for 10 min. After further washing, the slides were dehydrated with 70, 85 and 100 % ethanol, 2 min in each bath. The chromosomal DNA was denatured in 70 % formamide/2xSSC for 3 min at 72 °C. The slides were dehydrated again in a cold ethanol series (70, 85 and 100 %), 5 min each. The hybridization mixture, containing 100 ng of denatured probe, 10 mg/ml dextran sulfate, 2xSSC and 50 % formamide (final volume of 30 μl) were heated to95 °C for 10 min and then applied on the slides. Hybridization was performed for a period of 16–18 h at 37 °C in a moist chamber. After hybridization, the slides were washed for 5 min with 2xSSC and then rinsed quickly in 1xPBS. The detection of the probes was performed with Streptavidin-Cy3 (Sigma) for the 18S rDNA probe and anti-digoxigenin-FITC (Roche) for the 5S rDNA probe. The chromosomes were counterstained with DAPI (1.2 g/ml) in Antifading solution (Vector Laboratories).

### Microscopy analyses and image processing

Approximately 30 metaphase spreads were analyzed to confirm the diploid chromosome number, karyotype structure and FISH results. Images were captured on an Olympus BX50 microscope (Olympus Corporation, Ishikawa, Japan) using CoolSNAP and the Image Pro Plus 4.1 software (Media Cybernetics, Silver Spring, MD, USA). The chromosomes were classified as m or sm according to their arm ratios [61].

## Abbreviations

2n: diploid number
CMA_3_: Chromomycin A3
DAPI: 4',6-diamidino-2-phenylindole
dUTP: 2'-Deoxyuridine-5'-Triphosphate
FISH: Fluorescence *in situ* hybridization
M: Metacentric chromosome
NOR: Nucleolar organizing regions
PCR: Polymerase chain reaction
rDNA: ribosomal DNA
rRNA: ribosomal RNA
sm: submetacentric chromosome
